# Wound Care Knowledge, Attitudes, and Practices and Mobile Health Technology Use in the Home Environment: Cross-Sectional Survey of Social Network Users

**DOI:** 10.2196/15678

**Published:** 2020-03-26

**Authors:** Ya-Ting Kuan, Tze-Fang Wang, Chao-Yu Guo, Fu-In Tang, I-Ching Hou

**Affiliations:** 1 School of Nursing National Taipei University of Nursing and Health Sciences Taipei Taiwan; 2 School of Nursing National Yang-Ming University Taipei Taiwan; 3 Institute of Public Health National Yang-Ming University Taipei Taiwan

**Keywords:** mobile health, wound, knowledge, attitudes, practices, home environment

## Abstract

**Background:**

Injury causing wounds is a frequent event. Inadequate or inappropriate treatment of injuries can threaten individual health. However, little is known about wound care knowledge, attitudes, and practices and mobile health (mHealth) use in the home environment in Taiwan.

**Objective:**

This study aimed to evaluate wound care knowledge, attitudes, and practices and mHealth technology use among social network users.

**Methods:**

A cross-sectional survey on social media platforms was conducted on adults aged 20 years and older. Data were collected from social network users in the home environment.

**Results:**

A total of 361 participants were enrolled. The mHealth technology use of participants was positively correlated with wound care knowledge (*r*=.132, *P*=.01), attitudes (*r*=.239, *P*<.001), and practices (*r*=.132, *P*=.01). Participants did not have adequate knowledge (correct rate 69.1%) and were unfamiliar with the guidelines of proper wound care (correct rate 74.5%). Most participants had positive attitudes toward wound care and mHealth technology use. A total of 95.6% (345/361) of participants perceived that the use of mHealth technology can improve wound care outcomes, and 93.9% (339/361) perceived that wound care products should be optimized to be used with a mobile device. However, 93.6% (338/361) of participants had no experience using mHealth technology for wound care.

**Conclusions:**

Our study shows the potential of mHealth technology to enhance wound care knowledge among social network users. Thus, government agencies and medical institutions in Taiwan should provide easy-to-use information products that enhance wound care knowledge, promote adequate behavior toward wound care, and prevent unpredictable or undesirable outcomes.

## Introduction

Injuries causing wounds occur frequently. In 2015, up to 50 million people worldwide incurred injuries because of road traffic crashes resulting in additional indirect health consequences associated with this growing epidemic [1]. Inadequate or inappropriate treatment of injuries can threaten individual health. Injuries contribute to approximately 10% of mortality and 12% of morbidity worldwide [2]. In Taiwan, cases of road traffic accidents increased from 216,927 in 2007 to 403,906 in 2016, and this, in turn, resulted in an increase in the injury rate [3]. Similarly, in the United States, the number of road traffic accidents increased (from 2,491,000 in 2007 to 3,144,000 in 2016), and accordingly, the injury rate increased [4]. Wound care is one of the major challenges for health care systems [5,6] and accounts for 2% to 3% of the medical care budget [7]. Moreover, the demand for wound care is increasing. Patients who do not receive medical care or are discharged from medical institutions usually perform wound care by themselves at home. A study has found that 38.2% and 58.7% of patients returning from hospitals did not know how to change their dressing at home or how to clean the wounds, respectively [8]. Up to 84% of patients with surgical wounds return for regular follow-up [6]. If the wound is not properly treated, it may lead to infection (3% to 15%) [9,10]. Signs of wound infection include fever, swelling, pain, and purulent exudate. Factors such as corticosteroid use, smoking, and poor general health affect wound healing [11]. Patients who are not taught how to perform wound care and neglect the consequences of improper wound care experience a substantial economic burden and reduced quality of life [12].

Mobile health technology offers an alternative by increasing access to wound care resources [13,14] and involves the use of information and communication technology such as computers, mobile phones, personal digital assistants, and wearable sensors to deliver medical service and information [6,15]. In Taiwan, there were 29.31 million mobile communication users in 2019 [16]. The number of the population who are using a mobile phone to accessing internet is increase from 35.3% in 2011 to 88.2% in 2018 [17]. Mobile phone apps are an emerging tool for wound care [18]. Mobile health (mHealth) technology can support wound care knowledge, attitudes, and practices of social network users. Using mHealth technology (1) supports self-care among patients, (2) improves wound outcomes, (3) reduces care costs, (4) has built-in alerts, (5) enhances remote consultation, (6) promotes accurate assessment of wounds using wound images, (7) improves quality of life, and (8) supports nonspecialized caregivers [6,19-21]. Research has demonstrated that a lack of wound care knowledge and skills negatively affects the prognosis of wounds and health information technology use positively affects prognosis [8]. Users must be considered when designing health information technology [22]. Thus, wound care knowledge, attitudes, and practices and the mHealth technology use of social network users in Taiwan should be evaluated. However, little is known about these aspects. Therefore, this study aimed to evaluate the wound care knowledge, attitudes, and practices and mHealth technology use of individuals in the home environment in Taiwan. Findings regarding the use of mHealth technology among social network users were compared with prior work.

## Methods

### Study Design

We evaluated the wound care knowledge, attitudes, and practices and mHealth technology use of individuals in the home environment in Taiwan using a Web-based questionnaire survey. We conducted this Web-based questionnaire survey on a social media platform in the home environment between December 2015 and March 2016. Our subjects were Taiwanese individuals who had received wound care and sought health care information. A cross-sectional survey and network sampling were conducted to recruit subjects with background characteristics [23]. The study was approved by the National Yang-Ming University Human Research Ethics Committee (No YM104116E). A 4-part questionnaire was composed. Parts 1, 2, 3, and 4 were written to understand participant demographics, wound care experience, wound care competence, and mHealth technology use toward wounds, respectively. In the weeks preceding the formal investigation, a pretest was conducted in December 2015 with 31 subjects to ensure the clarity, conciseness, and readability of the scales and determine the approximate time required to complete the questionnaire. The formal investigation was anonymously conducted through a Web-based questionnaire survey addressed to participants in March 2016. Respondents were assured that their privacy was protected, and their informed consent was secured. To avoid duplicate responses, we only accepted the first response from the same internet protocol (IP) address without informing participants.

### Participants and Recruitment

**Figure 1:**
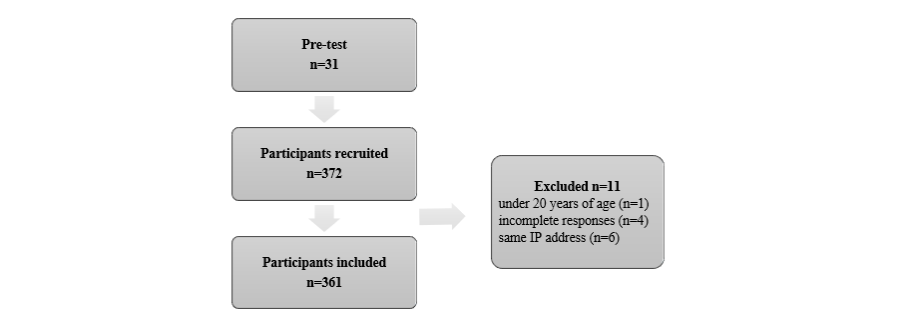
Flow chart of the study design and screening process.

Participants aged 20 years and older who had experience using computers, communication devices, or consumer electronics (the “3C” products) and the ability to understand and complete an online informed consent form were included. Participants were recruited in March 2016 through social media platforms including Facebook and Professional Technology Temple (PTT) pages created specifically for the survey. PTT is one of the largest social media platforms in Taiwan [24]. Participants who did not meet the inclusion criteria and those who submitted multiple responses were excluded. According to an instrumental study, the sample size should be greater than 300 if the target population is over 5000 [25]. This survey was conducted from March 4 to March 31, 2016, and garnered 372 responses, with 11 responses excluded (1: aged younger than 20 years, 4: incomplete responses, and 6: same IP address). A total of 361 responses were included in the data analysis (Figure 1). The effective response rate was 97.0%.

### Instrument

We developed an instrument and defined questions according to the literature reviews [5,8,11]. With regard to questions related to wound care knowledge and wound care practices, correct-error scales (10=correct, 0=error) were used. Answers were considered correct if the items selected followed the wound care guidelines [5,8,11]; otherwise, answers were considered incorrect. With regard to questions related to wound care attitudes and mHealth technology use, arithmetic scales (10=strongly agree, 7.5=agree, 5=neutral, 2.5=disagree, 0=strongly disagree) were used.

### Validity

To determine whether the instrument was appropriate and concise, we consulted wound care experts on content validity. Three experts—a licensed dermatologist working in a 2300-bed medical center; a nurse director at Taiwan Wound, Ostomy, and Continence Nurses Association; and an experienced nurse supervisor working in the burn center at a 1200-bed medical center—examined the entire instrument and offered suggestions and opinions on its content. These experts helped with appropriate wording and examined each item and the grouping. All the experts agreed that the four parts of the questionnaire were appropriate and clear. This questionnaire used a 4-point Likert scale with anchors ranging from 1=very inappropriate, 2=somewhat inappropriate, 3=appropriate, and 4=very appropriate and a 4-point Likert scale with anchors ranging from 1=very unclear, 2=somewhat unclear, 3=clear, and 4=very clear. The content validity index was 0.97, which was acceptable. After validation by the experts, the online instrument was created using Google Forms (Google LLC) and distributed to the enrolled participants.

### Reliability

In the pilot study, 31 participants were invited to complete the online instrument; this was used to examine whether the wound care knowledge, attitudes, and practices items and mHealth technology use items in the instrument had internal consistency. Cronbach alpha for the study was 0.72, which indicated acceptable internal consistency reliability [26].

The survey consisted of approximately 60 questions and took between 15 and 20 minutes to complete. Participants were permitted to discontinue the survey anytime. Participants who completed the survey and provided an email address received an NT $100 (US $3.32) gift card to a retail store. Only completed questionnaires were included in the analysis. Participant identifiers were removed from the survey before data analysis.

### Statistical Analysis

SPSS Statistics 23 software package (IBM Corporation) was used to analyze the data. Numbers, percentages, mean, standard deviation, and Pearson correlation (*r*) were used to examine the relationships between wound care knowledge, attitudes, and practices and mHealth technology use among the participants.

## Results

### Background Characteristics

A total of 361 social network users were included in this study. The main wound care knowledge resource among participants was health care professionals (367/1081, 34%), followed by experience/self-study (258/1081, 23.8%) and social media/internet/other (250/1081, 23.2%) (Table 1). Of participants, 60.7% (219/361) and 69.5% (251/361) had received medical treatment in the past 6 months and had incurred skin injuries in the previous year, respectively; 15.2% (55/361) were diagnosed with wound infection by a doctor; 24.4% (88/361) were not taught how to perform wound care; 33.2% (352/361) and 15.8% (342/361) used mobile phones and the internet, respectively; and while 78.1% (282/361) had been using mobile phones for more than 3 years, 93.6% (338/361) had never used the phone to look for wound care advice.

**Table 1:** Background characteristics of the participants (n=361).

Characteristic		Value
**Gender, n (%)**		
	Male	235 (65.1)
	Female	126 (34.9)
**Age in years, n (%)**		
	20-24	156 (43.2)
	25-29	114 (31.6)
	30-34	59 (16.3)
	≥35	32 (8.8)
Age in years, mean (SD)		26.5 (5.7)
**Education level, n (%)**		
	Associate degree	15 (4.1)
	Bachelor’s degree	270 (74.9)
	Master’s/doctorate degree	76 (21.0)
**Marital status, n (%)**		
	Married	37 (10.2)
	Not married	324 (89.8)
**Children, n (%)**		
	No	339 (93.9)
	Yes	22 (6.1)
**Wound care knowledge resource (select all that apply) (n=1081), n (%)**		
	School health education course	204 (18.9)
	Health care professionals	367 (34.0)
	Experience/self-study	258 (23.8)
	Social media/internet/other	250 (23.3)

### Wound Care Experience

The mean wound length and width were 4.02 (SD 5.71) cm and 2.40 (SD 2.73) cm, respectively. The primary causes of wounds were traffic accidents (124/361, 34.3%), penetrating injuries (59/361, 16.3%), and surgeries/diseases (58/361, 16.1%). A total of 48.5% (175/361) of participants incurred abrasion/contusion, 19.9% (72/361) incurred cuts, and 11.6% (42/361) other types of wounds. The primary causes of hospital visits were abrasions/contusions (184/606, 30.4%), lacerations (130/606, 21.5%), and cuts (105/606, 17.3%). Most of the participants incurred a wound on the knee (120/432, 27.8%), arm (87/432, 20.1%), or finger (86/432, 19.9%). Moreover, 89.8% (324/361) of participants applied the wound dressing by themselves, and 80.6% (291/361) had experience treating their own wound or treating others’ wounds at the hospital. Many participants (260/361, 72.0%) were afraid or lacked confidence in treating their own or others’ wounds. Wound care experience of the participants is presented in Table 2.

**Table 2:** Wound care experience of participants (n=361).

Characteristic		Value n (%)
**Primary cause of wound**		
	Traffic accident	124 (34.3)
	Penetrating injury	59 (16.3)
	Surgery/disease	58 (16.1)
	Falls	57 (15.8)
	Other/unclear	38 (10.5)
	Burn	13 (3.6)
	Bite/scratch	12 (3.3)
**Number of wounds**		
	1	226 (62.6)
	2	72 (19.9)
	3	31 (8.6)
	>3	32 (9.1)
**Type of wound**		
	Abrasion/contusion	175 (48.5)
	Cuts	72 (19.9)
	Other	42 (11.6)
	Laceration	32 (8.9)
	Unclear	24 (6.6)
	Scratch	7 (1.9)
	Insect bites	5 (1.4)
	Bruising	4 (1.1)
**Type of wound requiring hospital visit, select all that apply (n=606)**		
	Abrasion/contusion	184 (30.4)
	Laceration	130 (21.5)
	Cuts	105 (17.3)
	Insect bites	54 (8.9)
	Unclear	50 (8.3)
	Bruising	47 (7.8)
	Other	36 (6.0)
**Location of wound, select all that apply (n=432)**		
	Knee	120 (27.8)
	Arm	87 (20.1)
	Finger	86 (19.9)
	Leg	41 (9.5)
	Other	31 (7.2)
	Wrist	26 (6.0)
	Buttock	18 (4.2)
	Facial	13 (3.0)
	Head	10 (2.3)
**Wound appearance**		
	No sign of infection	122 (19.4)
	Dirty	97 (15.4)
	Partial thickness skin loss	181 (28.8)
	<10 cc bleeding	201 (32.0)
	>10 cc bleeding	28 (4.5)
**Type of wound disinfectant used, select all that apply (n=550)**		
	Povidone-iodine solution	242 (44.0)
	Normal saline solution	200 (36.4)
	Antibiotic ointment	74 (13.5)
	Mercurochrome/acrinol	23 (4.1)
	Other	11 (2.0)
**Type of wound dressing used, select all that apply (n=534)**		
	Gauze	216 (40.4)
	Adhesive bandage	135 (25.3)
	DuoDERM	107 (20.0)
	Antimicrobial dressing	30 (5.6)
	No dressing used	27 (5.1)
	Collagen dressing	6 (1.1)
	Chinese medicine dressing	6 (1.1)
	Other	7 (1.3)

### Wound Care Knowledge, Attitudes, and Practices

For wound care knowledge, more participants could correctly identify a photo of an abrasion wound than a diabetic foot or pressure ulcer wound. The percentage of participants with wound care knowledge was 69.1% (Table 3).

For wound care practices, most participants assessed wound appearance before dressing (339/361, 93.9%). Most participants washed their hands before the last wound dressing they performed (327/361, 90.6%). Less than one-quarter of participants (84/361, 22.4%) used a sterile cotton swab for wound dressing. The mean rate of correct wound care practices was 74.5% (Table 3).

For the wound care attitudes, 28.5% (103/361) of participants showed that they had good knowledge of assessing a wound (Table 4). Half of participants (183/361, 50.7%) worried about lacking ability to perceive wound infection or complication. Most participants (330/361, 91.5%) thought that the method of managing wounds was important for wound healing. However, only 27.1% (98/361) of participants had the confidence to care for wounds correctly.

**Table 3:** Wound care knowledge and practices (n=361).

Topic		Score^a^ (Correct %)		Rank	
**Wound care knowledge**					
	Identify image of an abrasion wound		9.9 (86.4^b^)		1
	Identify image of a diabetic foot		6.4 (63.7^c^)		2
	Identify image of a pressure ulcer wound		4.3 (42.7^d^)		3
	I believe not smoking promotes wound healing		10 (99.7)		1
	I believe nutrition may be a factor in promoting wound healing		9.4 (94.2)		2
	I believe getting enough sleep may be a factor in promoting wound healing		9.3 (93.4)		3
	I believe not using steroids may be a factor in promoting wound healing		7.6 (75.6)		4
	I believe keeping the moisture balance of the wound bed can help wound healing		5.8 (58.0)		5
	I believe appropriate exercise may be a factor in promoting wound healing		4.0 (40.1)		6
	I believe abnormal exudate may be a sign of wound infection		9.4 (93.6)		1
	I believe redness and swelling may be signs of wound infection		7.8 (77.6)		2
	I believe fever may be a sign of wound infection		6.8 (67.6)		3
	I believe pain may be a sign of wound infection		6.3 (63.4)		4
	I believe cold may be a sign of wound infection		1.2 (11.6)		5
**Wound care practices**					
	Assess wound appearance before dressing (eg, redness, exudate)		9.4 (93.9)		1
	Wash hands before dressing wound		9.1 (90.6)		3
	Remove gauze after rinsing wound with normal saline solution or boiled water		6.7 (67.1^e^)		7
	Use normal saline solution or boiled water to clean wound		7.0 (69.6^f^)		6
	Use sterile cotton swab to dress wound		2.2 (22.4^g^)		8
	Use dressing that covers wound margin by at least 1 cm all around		9.3 (92.8^h^)		2
	Contact position between the finger/clip and the dressing when covering		7.7 (76.8^i^)		5
	Wash hands after dressing wound		8.3 (82.9)		4

^a^Correct-error scales: 10=correct, 0=error.

^b^Bruising (0.8%), laceration (10.2%), cuts (0.3%), burns (1.9%), arteriovenous ulcer (0.3%).

^c^Bruising (2.5%), laceration (0.3%), abrasion/contusion (1.1%), burns (20.5%), arteriovenous ulcer (6.1%), pressure ulcer (5.8%).

^d^Bruising (3.6%), laceration (5.5%), cuts (0.6%), abrasion/contusion (10%), burns (13.6%), diabetic foot (17.7%), arteriovenous ulcer (6.4%).

^e^Removing sticking gauze directly (23.0%), removing gauze after rinsing with tap water (2.5%), removing gauze after rinsing with povidone-iodine solution (2.5%), removing gauze after rinsing with alcohol-iodine solution (2.8%), removing gauze after rinsing with hydrogen peroxide solution (1.1%), other (1%).

^f^No cleaning of wound (5.8%), tissue (7.2%), gauze (5.3%), tap water (10.2%), other (1.7%).

^g^Tissue (3.6%), gauze (15.5%), nonsterile cotton swab (22.4%), other (1.1%).

^h^Smaller than wound’s margin (2.2%); equal to wound’s margin (5%).

^i^The wound contact side of dressing (23.2%).

**Table 4:** Wound care attitudes (n=361).

Attitude	Negative (score 0, 2.5^a^) n (%)	Neutral (score 5^a^) n (%)	Positive (score 7.5, 10^a^) n (%)
I know very well how to assess wound.	63 (17.5)	195 (54.0)	103 (28.5)
I am worried about my lack of ability to perceive wound infection or complication.	183 (50.7)	110 (30.5)	68 (18.8)
I think that the method of managing a wound is important for wound healing.	6 (1.7)	25 (6.9)	330 (91.4)
I am confident I am doing wound care correctly.	77 (21.3)	186 (51.5)	98 (27.2)

^a^Arithmetic scale: 10=strongly agree, 7.5=agree, 5=neutral, 2.5=disagree, 0=strongly disagree.

### Mobile Health Technology Use

Most of the participants responded neutral to positively to the following (Table 5): it is important to use mHealth technology in wound care (345/361, 95.6%); the use of mHealth technology in wound care can be helpful in improving wound care outcomes (345/361, 95.6%); wound care information products should be optimized for mobile devices (339/361, 93.9%) and should be easy to use (341/361, 94.4%); and I am interested in how mHealth technology can help me take care of wounds (347/361, 96.1%). The mHealth technology use of participants was positively correlated with wound care knowledge (*r*=.132, *P*=.01), attitudes (*r*=.239, *P*<.001), and practices (*r*=.132, *P*=.01).

**Table 5:** Mobile health technology use of participants (n=361).

Content	Negative (score 0, 2.5^a^) n (%)	Neutral (score 5^a^) n (%)	Positive (score 7.5, 10^a^) n (%)
It is important to use mobile health technology in wound care.	16 (4.4)	164 (45.4)	181 (50.2)
The use of mobile health technology in wound care can be helpful in improving wound care outcomes.	16 (4.4)	109 (30.2)	236 (65.4)
Wound care information products should be optimized for mobile devices.	22 (6.1)	129 (35.7)	210 (58.2)
Wound care information products should be easy to use.	20 (5.6)	99 (27.4)	242 (67.0)
I am interested in how mobile health technology can help me take care of wounds.	14 (3.9)	92 (25.5)	255 (70.6)

^a^Arithmetic scale: 10=strongly agree, 7.5=agree, 5=neutral, 2.5=disagree, 0=strongly disagree.

## Discussion

### Principal Findings

This study showed that most participants (345/361, 95.6%) understood the importance of using mHealth technology and 96.1% (347/361) of participants were interested in using this method to help them take care of wounds at home. Most participants (324/361, 89.8%) had wound dressing experience, but participants lacked confidence in their ability to assess and perform wound care correctly (260/361, 72%). Most wounds can be self-managed at home [27]. Hence, patients should be taught how to perform basic wound assessment and determine any signs of complications and infections [8]. Most participants had a positive attitude toward wound care and mHealth technology use, but they did not have adequate knowledge (correct rate 69.1%) and were unfamiliar with the guidelines in performing proper wound care (correct rate 74.5%). Removing gauze sticking to the wound without wetting the gauze, which might harm granulating tissue, was not commonly done. A total of 30.4% of the participants did not use normal saline solution or boiled, sterilized water in cleaning the wound. The use of disinfectants to clean wounds with no sign of infection is not conducive to wound healing; hence, cleaning uninfected wounds with normal saline or boiled, sterilized water was suggested [11]. In addition, the sterile concept should be explained since patients used nonsterile cotton swabs, easily available at home, to clean wounds. Additionally, whether nonsterile cotton swabs can be used as an alternative to sterile cotton swabs in nonhospital settings should be further studied.

### Comparison With Prior Work

Wound care education can improve wound care attitudes and reduce the fear of taking care of wounds [5,10]. Participant wound care knowledge was positively correlated with wound care practices [28,29]. Thus, improving the wound care knowledge of the patient can enhance wound care skills [5]. Participants’ main resources for wound care knowledge were health care professionals. Other studies have found similar trends in which most social network users responded that contact with online professionals was somewhat important or very important [30]. Our study had similar results; in addition, we showed that participant wound care competency was positively correlated with mHealth technology use. Thus, policymakers should focus on increasing mHealth technology use and wound care competency among social network users in Taiwan. In addition, 95.6% of participants presumed that the application of mHealth technology can improve wound care outcomes, and 93.9% (339/361) perceived that wound care information products should be optimized to use with a mobile device. Developing software for mobile devices such as visual reality simulation for wound care to increase the interaction with wound care learners should be considered. In addition, developing artificial intelligence that can determine wound type or signs of infection is recommended, and a health care professional must be consulted to receive the correct care recommendations.

### Limitations

This study had some limitations. A single platform was used in selecting the sample. The findings of this study are limited to adults living in Taiwan, and the mean age was 26.5 years. Samples were not representative of all social network users in Taiwan as there were restrictions in patients’ age and wound type. Moreover, as self-report was adopted to understand the wound care knowledge, attitudes, and practices, it is possible that participants adhered to perceived social norms.

### Conclusion

Our study showed the potential of mHealth technology in enhancing wound care knowledge, attitudes, and practices among social network users in Taiwan. Most participants responded that it is important to apply mHealth technology in wound care. However, most of them had not used mHealth technology for wound care. Therefore, our results can serve as an important reference for conducting further studies on the use of mHealth technology in wound care among social network users in the home environment. The association among wound care knowledge, attitudes, and practices and mHealth technology suggests that government agencies and medical institutions should provide correct information for wound care knowledge to promote appropriate behavior toward wound care and prevent unpredictable or undesirable outcomes.

## Abbreviations

IP: internet protocol
mHealth: mobile health
PTT: Professional Technology Temple
